# The prevalence of hypercholesterolemia and associated risk factors in Al-Kharj population, Saudi Arabia: a cross-sectional survey

**DOI:** 10.1186/s12872-020-01825-2

**Published:** 2021-01-07

**Authors:** Jamaan Al-Zahrani, Mamdouh M. Shubair, Sameer Al-Ghamdi, Abdullah A. Alrasheed, Abdulrahman A. Alduraywish, Fayez Saud Alreshidi, Saeed Mastour Alshahrani, Majid Alsalamah, Badr F. Al-Khateeb, Aljawharah Ibraheem Ashathri, Ashraf El-Metwally, Khaled K. Aldossari

**Affiliations:** 1 Family and Community Medicine Department, College of Medicine, Prince Sattam Bin Abdulaziz University, Al-Kharj, 11942 Saudi Arabia; 2 School of Health Sciences, University of Northern British Columbia (UNBC), 3333 University Way, Prince George, BC V2N 4Z9 Canada; 3 Family and Community Medicine Department, College of Medicine, King Saud University, Riyadh, Saudi Arabia; 4 Internal Medicine Department, College of Medicine, Jouf University, Sakaka, Saudi Arabia; 5 Family and Community Medicine Department, College of Medicine, University of Hail, Hail, Saudi Arabia; 6 College of Applied Medical Sciences, King Khalid University, Abha, Saudi Arabia; 7 Department of Emergency Medicine, King Abdulaziz Medical City, College of Public Health and Health Informatics, King Saud Bin Abdulaziz University for Health Sciences, Riyadh, Saudi Arabia; 8 Department of Family Medicine, King Abdulaziz Medical City, College of Public Health and Health Informatics, King Saud Bin Abdulaziz University for Health Sciences, Riyadh, Saudi Arabia; 9 Clinical Nutrition, Community Health Department, Applied Medical Science, King Saud University, Riyadh, Saudi Arabia; 10 College of Public Health and Health Informatics, King Saud Bin Abdulaziz University for Health Sciences, Riyadh, Saudi Arabia

**Keywords:** Hypercholesterolemia, Obesity, Cross-sectional, Al-kharj, Saudi arabia

## Abstract

**Background:**

Hypercholesterolemia (HC) is an important precursor to many cardiovascular, cerebrovascular, and peripheral vascular diseases. A report conducted by the American Heart Association showed the prevalence of HC to be 11.9%, with around 28.5 million adults age ≥ 20 years having high cholesterol levels. This study aimed to evaluate the prevalence of HC and its associated risk factors among the general population of Al-Kharj, Saudi Arabia.

**Method:**

A cross-sectional study was conducted on the general population of Al-Kharj, Saudi Arabia in 2016. The representative sample consisted of 1019 individuals, who all participated on a voluntary basis. The statistical analysis was performed using SPSS version 25.

**Results:**

The results of this study showed the prevalence of HC in the sample to be 12.5%. There was a significant moderate positive association between increasing age and the prevalence of HC (r = 0.240, *P* < 0.0001). Males had a significantly higher prevalence of HC (56.7%) compared to their female counterparts (43.3%) (*X*^2^ = 23.093, *P* ≤ 0.0001). BMI was positively and significantly associated with high cholesterol status. Participants in the overweight category had a significantly higher risk of HC (OR = 1.727; 95% CI = 1.58–1.914; *P* = 0.046). The non-obese (< 25 kg/m^2^) participants had an inverse significant association with the risk of hypercholesterolemia. (OR = 0.411; 95% CI = 0.216–0.783; *P* = 0.007).

**Conclusion:**

In this population-based study, the predominant risk factors of HC in Al-Kharj region were being of a Saudi nationality, male, having obesity, being unemployed, and being a civilian worker. There is a clear need for future screening studies of HC, as most previous studies have reported contradictory prevalence data (because they were conducted in different regions of KSA). Furthermore, well-designed prospective cohort studies are needed in the future to assess how the association between lifestyle behavioural factors such as dietary intake patterns and levels of physical activity may affect the relative risk of HC status.

## Background

Elevated blood cholesterol level better known as hypercholesterolemia (HC) is an well-established risk factor for cardiovascular, cerebrovascular, and peripheral vascular diseases [1]. Hypercholesterolemia can be either due to primary (genetic or familial), or secondary (acquired) causes. Genetic mutations of the LDL receptor gene account for 85% of familial causes. Other factors include defective apolipoprotein B, proprotein convertase subtilisin/kexin type 9 gene gain-of-function mutation, LDL receptor adaptor protein mutation and polygenic HC [2–4]. Acquired causes include medical conditions which include hypothyroidism, diabetes mellitus, nephrotic syndrome, and cholestasis. Some medications such as cyclosporine and thiazide diuretics, as well as excessive intake of dietary cholesterol and smoking have been linked with increased risk of HC [1, 5, 6].

Age is also a strong risk factor for HC; more advanced age has been associated with higher risk of HC for both sexs [7–9]. In people who are in younger age groups (teenagers, young adults 18–25 years of age), HC can go undetected [10]. In a study by Basulaiman et al., it was reported that 65% of Saudis with HC were undiagnosed [9]. Another study reported that the prevalence of HC was 23.7% in the young age group [10]. There is currently a gap in the literature when it comes to HC, as there is a very limited number of studies which include young adults (18–25 years of age) [9, 11, 12]. This age group seems to be at a relatively lower risk of having HC; however, according to the National Cholesterol Education Program (NCEP), adults 20 years of age and older should be screened for HC at least once every 5 years [13]. Additionally, in a surprising cross-sectional study conducted in children (9–12 years)—not even young adults—in Riyadh showed a prevalence of HC of 32.7% [14].

Sex is a risk factor for HC which requires more extensive research. While some studies reported greater numbers of HC among males [7, 8], other investigations have on the contrary reported higher levels in females [12, 15]. Furthermore, there were other ‘unusual’ factors that showed some association with HC, including insomnia and early menopause [16, 17].

Globally, the prevalence of HC is relatively high. The World Health Organization (WHO) reported a global prevalence of 39% in 2008 [18]. Recent estimates have shown that around 28.5 million people from the adult population (aged 20 years or older) have high levels of total serum cholesterol, with the reported prevalence being 11.9% [19]. Data on HC levels in the Middle East are very limited. In a study conducted in 14 African and Middle Eastern (AFME) countries, the dyslipidemia level was as high as 70%. However, the prevalence of HC was not mentioned [20]. In Saudi Arabia, the prevalence of HC ranges widely from 8.5 to 54.9% in both the general population and the stable clinic visitors in different regions of the Kingdom [11, 12, 21].

This wide range of HC prevalence figures could be due to the fact that HC is an asymptomatic condition. It could also be a result of the differences between the studied age groups, sex, ethnic heterogeneity, and many other factors. Given that studies on HC are scarce in the Middle East and in Saudi Arabia, there is a significant need for a thorough cross-sectional population-based study in the region. Therefore, the current study aims to provide reliable data on the prevalence of hypercholesteremia at a population level. In order to have a comprehensive view of the topic, the study investigated the relationship between hypercholesteremia and its associated factors, including age, sex, marital status, body mass index (BMI), education level, employment status, diabetes status, and smoking status.

## Methods

This was a cross-sectional study conducted in Al-Kharj region of Saudi Arabia. The study’s duration was six months, starting from January to June 2016. The estimated total population of the city at the time of the study was reported to be slightly over 0.3 million (n = 300,000). Al-Kharj is one of the Kingdom’s major hubs, having great economic importance and up-to-date administration. It also possesses significant natural resources, an important geographical location, and population diversity (a population with a variety of ethnic backgrounds). Moreover, published studies showed that among the population of Al-Kharj, the prevalence of hypertension was 30.3%, the prevalence of obesity was 49.6%, and the risk of coronary heart disease (CHD) was 2.5% [22]. Given the increased prevalence of these chronic conditions (hypertension, obesity, and CHD), the assessment of hypercholesterolemia and its association to certain risk factors for CHD in the current study is warranted.

### Study population

**Fig. 1:**
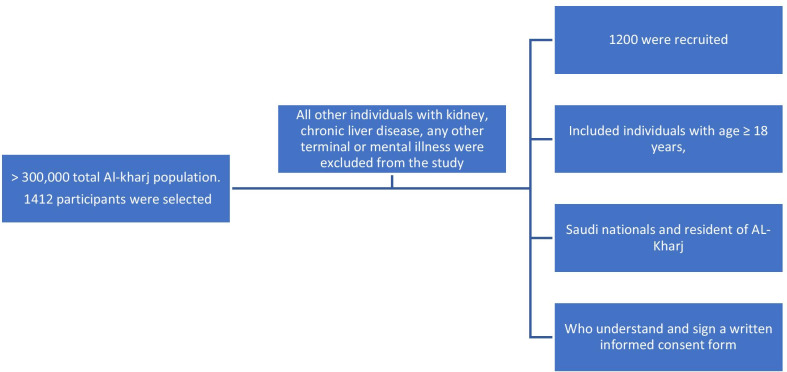
Flowchart of inclusion and exclusion criteria

Figure 1 represents the inclusion and exclusion criteria of the study population.

### Data collection procedure and tools

A total of 1200 respondents participated in the study, with a response rate of 85%. All incomplete questionnaires in which more than 5 responses were missing were excluded. In the final analysis, the data of a total of 1019 respondents were included. A multistage sampling technique was utilized for the collection of the data. Samples were selected from different governmental and private institutions through a cluster sampling technique. Afterwards, cluster lists were made, and seven clusters were randomly selected by the investigators for inclusion in the study. Another round of cluster sampling was performed on the selected cluster, and two clusters of that were then included. The total population of the selected clusters was then divided into different sampling units using the list of participants obtained from each department of the selected institutes. Study respondents were then selected using simple random sampling from each of the selected groups through a computer-generated list. Finally, the eligible subjects among them were recruited in the study. The aforementioned sample population has also been used in previously published studies [23–25].

### Material and measures

Data were collected from the participants using a structured questionnaire. The questionnaire used in this study has been published previously [23, 26]. Information about sociodemographic factors such as age, sex, marital status, and education levels was obtained via the same questionnaire. Anthropometric measurements like body weight (kilograms), height (meters), Body Mass Index (BMI) and waist circumference (centimeters) were taken at the time of the interview by trained nurses. All the procedures were performed based on the standards for anthropometric measurements. In addition to this, a blood sample was drawn for a fasting (10–12 h fast) lipid profile from each respondent.

### Operational definition

Hypercholesterolemia (HC) is defined as a blood cholesterol level of > 200 mg/dL or > 5 mmol/L [27]. Non-obese or normal weight is defined as a BMI of < 25 kg/m^2^, and overweight denotes a BMI of ≥ 25 kg/m^2^, while a BMI of ≥ 30 kg/m^2^ falls within the obese category [28].

### Data analyses

Data were analyzed using SPSS version 25.0 (SPSS Inc., Chicago, Illinois, USA) for Windows. A simple chi-square test was used to examine the association between HC and different categorical variables. Univariate analysis was conducted using the bivariate correlation coefficient (Pearson’s *r*), while a multinomial regression model was used to test the relationship (adjusted odds ratio) between HC and all other possible risk factors. The confidence interval was 95%, and a *P* value of less than 0.05 was set to establish a statistical significance.

## Results

**Fig. 2:**
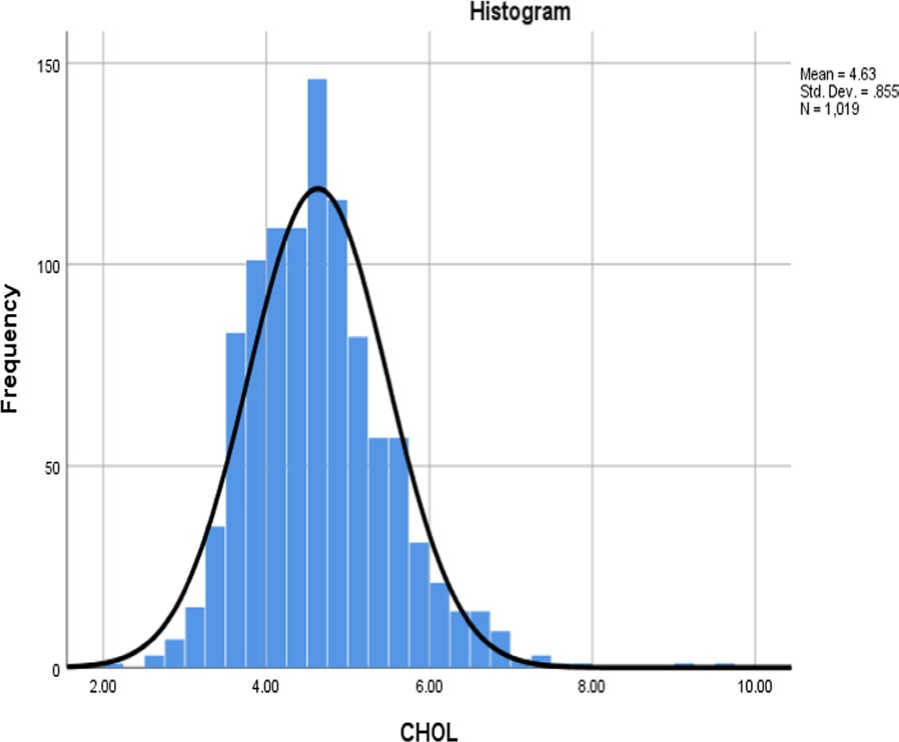
Normal distribution of serum cholesterol in Al-Kharj population (n = 1019)

Data related to the description of the study population were previously published [23, 26]. Figure 2 shows the normal distribution (Bell curve/Histogram) of the original continuous serum cholesterol variable, with a mean of 4.63 mmol/L and a standard deviation (SD) of 0.855 for the entire sample (n = 1019). There were no outliers in the normal distribution of the serum cholesterol levels.

### Univariate analysis

Bivariate correlation coefficient (Pearson’s r) was conducted between age of respondents.

(continuous variable) and binary HC status (1 = no HC; 2 = yes.

hypercholesterolemia).

The bivariate correlation coefficient (Pearson’s *r*) was conducted between the ages of the respondents (continuous variable) and binary HC status (1 = no HC; 2 = yes HC). The results showed that there was a significant moderate positive association between increasing age and the prevalence of HC (r = 0.240, *P* < 0.0001). Another Pearson’s *r* correlation coefficient test revealed that a high body mass index (BMI), as a continuous variable, was positively and significantly associated with the presence of HC (r = 0.181, *P* < 0.0001).

The prevalence of HC as a binary variable (1 = no HC; 2 = yes HC) was contrasted with sociodemographic and other risk factors. Seven (n = 7) cross-tabulation (*X*^2^) tests were conducted to assess the relationship between HC status and each of: sex, marital status, educational attainment, job type, diabetes status, smoking status, and body mass index (BMI) class/category.

The results are summarized in Table 1. A higher proportion of males (56.7%) had a significantly higher prevalence of HC compared with their female counterparts (43.3%). The Chi-Squared test was (*X*^2^) = 23.093, *P* ≤ 0.0001. Married individuals were significantly more likely to have HC (67.7%) in comparison to individuals who had never married (32.2%). The Chi-Squared test was (*X*^2^) = 68.086, *P* ≤ 0.0001. When HC status was examined according to educational attainment, it was found that secondary school students (23.6%) and university students (63.8%) had the highest prevalence of HC. The Chi-Squared test was (*X*^2^) = 27.177, *P* ≤ 0.000. In regard to job/employment type, a significant proportion of civilian workers had HC (84.3%) compared with those who were unemployed or were soldiers. The Chi-Squared test was (*X*^2^) = 56.683, *P* ≤ 0.0001.

**Table 1:** Univariate analysis regarding HC status in the Al Kharj population sample (n = 1019)

Variables	Hypercholesterolemia		Total (n = 1019)	Chi-square (*X*^2^)	*P* value
	No (n = 892)	Yes (n = 127)			
	n (%)	n (%)			
Respondent age	Mean (SD)		Mean (SD)	–	–
	25.6 years (8.6)	31.8 years (8.6)	26.4 years (8.6)		
Respondent sex					
Male	309 (34.6)	72 (56.7)	381 (37.4)	23.093	≤ 0.0001
Female	583 (65.4)	55 (43.3)	638 (62.6)		
Marital status					
Not married	621 (69.6)	41 (32.3)	662 (65.0)	68.086	≤ 0.0001
Married	271 (30.4)	86 (67.7)	357 (35.0)		
Education level					
Primary	15 (1.7)	7 (5.5)	22 (2.2)	27.177	≤ 0.0001
Secondary	101 (11.3)	30 (23.6)	131 (12.9)		
Intermediate	28 (3.1)	2 (1.6)	30 (2.9)		
University	719 (80.6)	81 (63.8)	800 (78.5)		
Postgraduate	29 (3.8)	7 (5.5)	36 (3.5)		
Job type					
Not working	28 (3.1)	4 (3.1)	32 (3.1)	56.683	≤ 0.0001
Civilian	440 (49.3)	107 (84.3)	547 (53.7)		
Soldier	424 (47.5)	16 (12.6)	440 (43.2)		
Diabetes status					
Diabetic	858 (96.2)	116 (91.3)	974 (95.6)	6.195	0.013
Non-diabetic	34 (3.8)	11 (8.7)	45 (4.4)		
Smoking status					
Non-smoker	787 (88.2)	100 (78.7)	887 (87.0)	9.044	0.011
Ex-smoker	24 (2.7)	7 (5.5)	31 (3.0)		
Current smoker	81 (9.1)	20 (15.7)	101 (9.9)		
BMI class					
Non-obese (< 25 kg/m^2^)	439 (49.3)	26 (20.5)	465 (45.7)	41.090	≤ 0.0001
Overweight (25–29.9 kg/m^2^)	230 (25.8)	42 (33.1)	272 (26.7)		
Class I obese (30–34.9 kg/m^2^)	134 (15.0)	35 (27.6)	169 (16.6)		
Class II/III obese (≥ 35 kg/m^2^)	88 (9.9)	24 (18.9)	112 (11.0)		

The relationship between HC and diabetes status showed that individuals with diabetes had a higher prevalence of HC (91.3%) than non-diabetic individuals with HC (8.7%). The Chi-Squared test was (*X*^2^) = 6.195, *P* = 0.013 (Table 1). There was a significant increase in the proportion of current smokers who had high cholesterol levels (15.7%), as opposed to only 5.5% of ex-smokers who had high cholesterol levels. The Chi-Squared test was (*X*^2^) = 9.044, *P* ≤ 0.011. Across the BMI categories, the results revealed that the overweight category showed a significantly higher proportion (33.1%) which had high cholesterol levels, followed by the class I obese category (27.6%), and then the class II/III obese group (18.9%). The Chi-Squared test was (*X*^2^) = 41.090, *P* ≤ 0.0001.

### Multiple logistic regression analyses

A first multiple logistic regression analysis was conducted by using the dichotomous HC status (outcome variable) with BMI (continuous independent variable) and other sociodemographic and lifestyle risk factors (Table 2). The results showed that BMI was positively and significantly associated with high cholesterol status. The odds ratio (OR) was 1.64 (95% CI = 1.610–1.671; *P* = 0.008). Marital status showed a significant inverse association with HC, in that married individuals had a significantly lower risk of having high cholesterol levels. The OR was 0.396 (95% CI = 0.236–0.664; *P* ≤ 0.0001). Moreover, job type/status—whether ‘not working’ (unemployed) or ‘civilian’—was positively and significantly associated with HC. The OR for the ‘not working’ status was 3.988 (95% CI = 2.051–5.139; *P* = 0.042), while the OR for a ‘civilian’ job status was 3.385 (95% CI = 1.722–6.652; *P* ≤ 0.0001).

**Table 2:** Logistic regression model using BMI (continuous variable) as a predictor for hypercholesterolemia status (binary outcome), after adjusting for sociodemographic and other variables (n = 1019)

Hypercholesterolemia status	B	SE of B	*P* value	Exp (B)/odds ratio	95% CI for odds ratio	
					Lower	Upper
Body Mass Index (BMI)	2.039	0.015	0.008	1.640	1.610	1.671
Age	0.012	0.016	0.452	1.012	0.981	1.044
Sex (female)	− 0.288	0.258	0.029	0.760	0.452	1.248
Marital status (married)	− 0.926	0.263	0.0001	0.396	0.236	0.664
Education level	0.379	0.671	0.572	1.461	0.392	5.440
Job (not working)	1.383	0.681	0.042	3.988	2.051	5.139
Job (civilian)	1.219	0.345	0.0001	3.385	1.722	6.652
Diabetes (yes)	0.026	0.408	0.950	1.026	0.461	2.284
Smoking status (no)	− 0.170	0.311	0.584	0.843	0.459	1.551
Smoking status (ex-smoker)	0.188	0.521	0.719	1.207	0.434	3.352

B = Beta Coefficient, SE of B = standard error of beta coefficient

A second multiple logistic regression analysis was further carried out to examine the association between BMI class (categorical variable) and the relative risk of HC, after adjusting for sociodemographic and lifestyle risk factors (Table 3). The results showed that overweight respondents had a significantly higher risk of HC. The odds ratio (OR) was 1.727 (95% CI = 1.58–1.914; *P* = 0.046). Non-obese (< 25 kg/m^2^) participants had an inverse significant association with the risk of HC. The OR was 0.411 (95% CI = 0.216–0.783; *P* = 0.007). Marital status and job type/status were also significantly inversely and positively associated with HC respectively (Table 3).

**Table 3:** Logistic regression model using BMI class (categorical variable) as a predictor for hypercholesterolemia status (binary outcome), after adjusting for sociodemographic and other variables (n = 1019)

Hypercholesterolemia status	B	SE of B	*P* value	Exp (B)/odds ratio	95% CI for odds ratio	
					Lower	Upper
BMI class (non-obese)	− 0.889	0.329	0.007	0.411	0.216	0.783
BMI class (overweight)	1.319	0.973	0.046	1.727	1.58	1.914
Age	0.010	0.016	0.512	1.010	0.980	1.042
Sex (female)	− 0.275	0.258	0.026	0.759	0.458	1.259
Marital status (married)	− 0.883	0.268	0.001	0.413	0.245	0.698
Education level	0.447	0.672	0.506	1.563	0.419	5.833
Job (not working)	1.394	0.684	0.042	4.030	2.054	5.415
Job (civilian)	1.217	0.346	0.000	3.377	1.713	6.656
Diabetes (yes)	0.042	0.412	0.920	1.042	0.465	2.335
Smoking status (no)	− 0.140	0.312	0.653	0.869	0.471	1.602
Smoking status (ex-smoker)	0.189	0.524	0.718	1.209	0.432	3.377

B = beta coefficient, SE of B = standard error of beta coefficient

## Discussion

The study findings show that increasing age and high BMI have significant positive associations with HC. While evaluating sociodemographic and other risk factors, it was found that HC was significantly associated with being male, married, a university graduate, a civilian worker, diabetic, a smoker, overweight or obese. On the other hand, in the multiple regression analysis, being overweight, unemployed and being a civilian worker were significant positive predictors. Whereas non-obese (< 25 kg/m^2^) and married individuals had a significant inverse association with HC.

The prevalence of HC in this study population is around 12.5%. A recent study conducted in Al-Hofuf region showed findings that were nearly consistent with those of this analysis [15]. In previously published studies with large sample sizes, it was reported that the prevalence of HC was 54% (in 2008) and 8.5% (in 2013) among Saudis [7, 9]. However, the prevalence of HC varies between different populations. The prevalence of HC appears to be increasing over time in developing countries [29]. For instance, the reported prevalence among the Nigerian population is 38% [30], while Taiwan has an estimated prevalence of 44% [31], and Korea reported a prevalence of 19.5% in 2015 [32]. Data from WHO’s Global Health Observatory showed that HC was highest in Europe (54%), followed by America (48%), South East Asia (29%), and Africa (22.6%) [18].

It was found that males had a higher prevalence of HC than females. Likewise, a recent university survey reported that HC and hypertriglyceridemia were more common among males [8]. The recent Africa Middle East Cardiovascular Epidemiological (ACE) Study reported significant differences between males (81%) and females (63%) [20]. In a Japanese prospective cohort study, unemployed men with fewer than 13 years of education had higher risk of HC [33]. However, contrasting results were found in some studies, in which HC was more frequently found in females [7, 30, 34], while other studies reported no differences in the prevalence of HC of both sexs [7].

Similarly, being overweight, unemployed, and being a civilian worker were positively associated with HC. Another Saudi study reported a higher prevalence of HC, with the main cause being a combination of unhealthy dietary habits and a lack of physical activity [7]. In one study, the estimated prevalence of physical inactivity was 96%, which increased with age and decreased with higher education level [35]. The key drivers for these findings are urbanization and the ubiquity of unhealthy diets and processed food across the entire Kingdom of Saudi Arabia [36]. Nonetheless, one study found no association between BMI and HC [29].

Furthermore, the current study found a higher prevalence of hypercholesterolemia among diabetic participants. A recently published Berlin Aging Study II (BASEII) found that elderly individuals on lipid lowering medication had a higher prevalence of diabetes [10]. However, most published studies have reported a higher prevalence of HC among diabetic individuals [27, 37]. A study conducted in Jeddah among primary healthcare employees reported that diabetic participants had a significant nine-fold increased risk of developing HC (OR = 9.27; 95% CI = 1.68–52.19, *P* = 0.019)[29]. Another Saudi study reported that the risk of having HC was doubled for diabetic individuals [9].

Naturally, there were few limitations to the current study. First, this was a cross-sectional study; therefore, causal inferences for observed significant associations cannot be made. Second, some risk factors were not explored, such as dietary habits, daily consumption of fat, physical activity/inactivity, family history, and drug/medication history, which could all be plausible contributory factors impacting the prevalence of HC. Third, the findings of the current study cannot be generalizable to some other countries due to differences in geographic location, lifestyle factors, and medical healthcare facilities. On the other hand, the major strength of the current study is its utilization of cluster sampling techniques, which, according to WHO’s recommendation, assures that the recruited sample accurately represents the entire population. The study also included common risk factors and evaluated their association with HC. Hypercholesterolemia (HC) and BMI were not self-reported but were measured through blood samples, while height, weight and all other data were collected by trained staff to ensure the reliability of the data.

## Conclusion

In a population-based study, the prevalence of HC in Saudi Arabia was found to be 12.5%, which is lower than what was reported in previous studies. Regarding the predominant risk factors, it was found that being Saudi, male, having obesity, being unemployed, and being a civilian worker were factors associated with HC in Al-Kharj region. These findings indicate that many non-communicable diseases, particularly cardiovascular disease (CVD), can be prevented by targeting high risk populations. Community-based awareness and education campaigns should be directed towards increasing individuals’ knowledge of the risk factors for HC. There is also a need for more screening studies, as most of the studies conducted in different regions of KSA show different prevalence rates. Furthermore, well-designed prospective cohort studies are warranted in the future in order to assess how the relationship between dietary intake patterns and physical activity/inactivity levels my affect the risk of HC.

## Data Availability

The data used in this study is only available upon request by the corresponding author of Prince Sattam Bin Abdulaziz University.
